# Development and Validation of a Nurse‐Led Developmental Intervention Package to Improve the Neurodevelopmental Outcomes of Preterm Babies in Neonatal Intensive Care Unit

**DOI:** 10.1002/pdi3.70016

**Published:** 2025-09-14

**Authors:** V. A. Raghu, Manju Vatsa, Neeraj Gupta, Hansraj, Ramesh Choudhary

**Affiliations:** ^1^ College of Nursing AIIMS Jodhpur Rajasthan India; ^2^ College of Nursing AIIMS New Delhi India; ^3^ Neonatology Department AIIMS Jodhpur Rajasthan India; ^4^ NICU AIIMS Jodhpur Rajasthan India

**Keywords:** developmental supportive care (DSC), neurodevelopment, nurse‐led, early intervention, preterm

## Abstract

Preterm birth is a major global health concern associated with high morbidity and mortality rates, along with long‐term neurodevelopmental impairments. Developmental supportive care (DSC) plays a crucial role in optimizing neurodevelopmental outcomes in these vulnerable neonates. This study aimed to develop, validate, and assess the feasibility of a nurse‐led developmental intervention package (NLDIP). A Medical Research Council (MRC) framework was used to develop and validate the NLDIP. The NLDIP was designed based on a comprehensive scoping review, expert consultations, and practical feasibility assessments in a neonatal intensive care unit (NICU). Validation was conducted through single‐round Delphi technique with expert reviews from different fields. The content validity index value was 0.9 and a dry run was performed to assess compliance and feasibility. The NLDIP was systematically developed and validated, comprising three key components: standard DSC, age‐appropriate multisensory stimulation (MSS) during hospitalization, and continued MSS at home. The findings demonstrate that the NLDIP is a valid, feasible, and well‐structured intervention which can be effectively implemented by nurses in an integrated way to enhance the neurodevelopmental outcomes of preterm infants.

## Graphical abstract

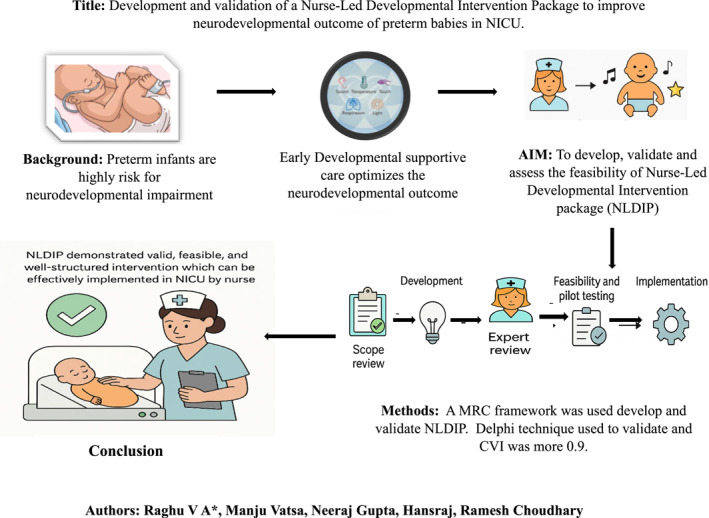

## 1 Introduction

Preterm birth is a major global health issue, with over 15 million cases annually, accounting for more than 10% of births [1]. Complications contribute to over 1 million deaths yearly, many of which are preventable through cost‐effective interventions [2]. India, with the highest number of preterm births (3.5 million), faces long‐term neurodevelopmental impairments in survivors, highlighting the need for targeted interventions to improve outcomes [3].

Preterm infants are neurologically immature and physiologically unstable, making them vulnerable to developmental delays [4]. The NICU environment, while essential for survival, can expose them to excessive and inappropriate sensory stimuli, negatively affecting their neurodevelopment [5]. Studies show that very low birth weight (VLBW) and extremely low birth weight (ELBW) infants face significant cognitive, motor, and socio‐emotional delays that can persist into later life [6, 7].

Developmental supportive care (DSC) can reduce these risks through structured interventions delivered to preterm infants to meet the individualized needs [8]. Research indicates that early developmental interventions including multisensory stimulation positively impact neurodevelopment, in terms of motor function, and cognition of preterm babies [9]. However, a standardized, nurse‐led intervention package is lacking, particularly in resource‐limited settings where specialized neonatal services are scarce. The literature review highlighted a research gap that necessitates future studies of large‐scale, integrated developmental supportive care for preterm babies. None of the studies used combined interventions, which are uniquely lead by the nurses in the NICU.

A nurse‐led developmental intervention package (NLDIP) is crucial to bridging gaps in neonatal care and improving neurodevelopmental outcomes. Nurses, as primary NICU caregivers, are well‐positioned to administer early interventions, ensuring consistency and quality while supporting both infants and families. Standardized programs can enhance care delivery and long‐term developmental trajectories.

A structured, evidence‐based, nurse‐led intervention provides a cost‐effective and sustainable approach to improving outcomes, particularly where specialized neurodevelopmental services are limited. Research highlights the benefits of early interventions that mimic intrauterine sensory experiences in enhancing cognitive and motor development. However, there is limited research on developing and validating a comprehensive, nurse‐led intervention tailored to preterm neonates. The author assumes that the integration of developmental supportive care with multisensory stimulation (MSS) during hospitalization and continuation of MSS at home setting as a unified package could be more beneficial than practicing few DSC intervention in NICU. Hence, this study aims to develop and validate a nurse‐led intervention package to enhance neurodevelopmental outcomes in preterm infants. By integrating evidence‐based developmental strategies and leveraging neonatal nurses' expertise, the intervention seeks to promote brain growth, prevent delays, and improve overall development. Findings will contribute to neonatal care knowledge and provide a framework for implementing nurse‐led interventions in diverse healthcare settings.

## 2 Materials and Methods

**FIGURE 1:**
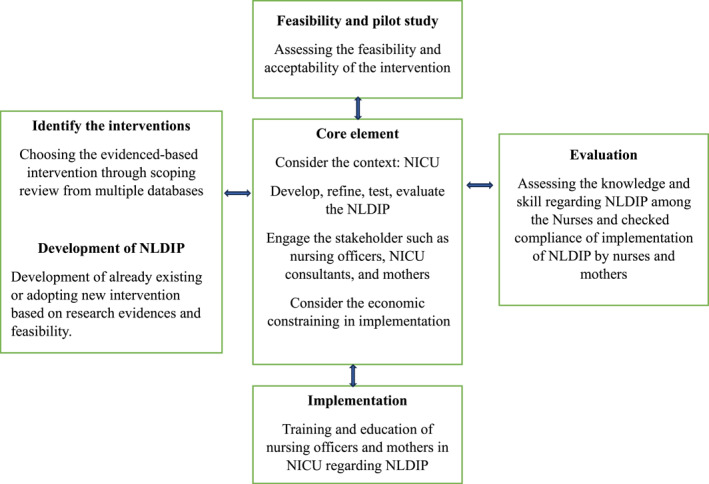
MRC framework for the development of nurse‐led developmental intervention package. NICU, neonatal intensive care unit; NLDIP, nurse‐led developmental intervention package.

The MRC framework comprises four interconnected phases: “development,” “feasibility and piloting,” “evaluation,” and “implementation” of a complex intervention [10]. This study adopted all four phases of the MRC framework to guide and development of the NLDIP (Figure 1). The development phase of intervention in the MRC framework contains three steps: identifying the evidence base, identifying/developing theory, and modeling the process and outcomes [10]. In this study, only first and second steps were included. The NLDIP was developed based on the comprehensive scoping review from multiple databases. An appropriate search strategy was used to extract the evidence‐based developmental supportive interventions from many databases. Publications were included with only English language and no restrictions on the publication period. This search yielded 21 studies; most of the studies were systemic reviews and meta‐analyses and randomized controlled trials (RCTs), which facilitated the identification of developmentally supportive intervention for preterm babies. These findings provided the foundational basis for the development of the NLDIP. In the second step, we explored the relevant and guiding theory that would enhance the effectiveness of the NLDIP. The initial interventions were derived based on the theoretical model such as synactive theory, transactional model, Newborn Individualized Developmental Care and Assessment Program (NIDCAP), and the neonatal integrative developmental care model [9, 11, 12]. A descriptive design utilizing a one round Delphi technique was used in this study to validate the NLDIP from experts from the different fields. The feasibility and pilot study were conducted to assess the feasibility of implementation of interventions which include pretesting, testing, and compliance checking. NLDIP was implemented in NICU through training nursing officers and mothers of preterm babies. Finally, the intervention package was evaluated for its feasibility of implementation in NICU and at home.

## 3 Result

### 3.1 Phase 1: Development of NLDIP

#### 3.1.1 Identifying the Evidence Base

To identify existing evidence regarding the effectiveness of developmental supportive intervention for enhancing the neurodevelopment outcome of preterm babies, we performed a comprehensive search of multiple databases, including Medline, CINAHL, PubMed, Scopus, Google scholar, and Research gate. The PICO format (population, intervention, comparison, and outcome) guided the search for relevant national and international studies. Medical Subject Headings (MeSH) terms utilized included “preterm,” “premature,” “neonate,” “early intervention,” “NICU,” “developmental supportive care,” “neurodevelopment,” and “multisensory stimulation,” and related keywords were used to extract relevant articles. Boolean operators and advanced search strategies were employed to optimize the search. We identified a total of 154 articles, out of which 21 studies were ultimately included (Table 1), and analyzed for the effectiveness of early developmental intervention on neurodevelopment outcomes.

**TABLE 1:** Study characteristics of selected studies for the development of NLDIP.

Author and year	Objective	Methodology	Intervention	Key findings
Carneiro et al. (2024) [13]	Nest positioning on motor development, sleep pattern, and weight gain	Systemic review	Nesting and therapeutic position in NICU	Evidence suggests nesting position improves the motor development and sleep quality in preterm babies in NICU
Queirós et al. (2023) [14]	Nonpharmacological management of neonatal pain	Systemic review	Sucrose/glucose solutions, NNS, breastfeeding, olfactive stimulus, auditory stimulus and sensory stimulus, KMC, therapeutic massage, and swaddling/facilitated tucking	Evidence suggests nonpharmacological approaches are safe, effective, and can be easily applied in daily practice and effective in reducing the neonatal pain
Yue et al. (2021) [15]	Effects of music therapy on preterm neonates	Systematic review & meta‐analysis	Music therapy	Improved HR, RR, and feeding volume; no effect on oxygen saturation
Johnston et al. (2021) [16]	Develop Brazilian guidelines on MSS in NICU	Systematic review	MSS	Supports unimodal and multimodal MSS to improve weight gain, sucking, and vital signs
Aita et al. (2021) [17]	Assess effectiveness of interventions on early neurodevelopment in preterm infants	Systematic review & meta‐analysis	Interventions: NIDCAP, DSC, sensory stimulation, parental participation, music therapy, and physical therapy	NIDCAP improved neurodevelopment
Khurana et al. 2020 [18]	Effect of neonatal therapy on the motor, cognitive, and behavioral development of infants born preterm.	Systematic review	Parent‐delivered motor intervention Therapist‐delivered postural control intervention DSC Oro‐motor intervention	Parent‐delivered motor interventions are effective than other interventions. The DSC program designed by therapist was effective in improving the short‐term behavior
Spittle et al. 2015 [19]	To evaluate early developmental interventions for preterm infants	Systematic review & meta‐analysis	Developmental intervention in NICU	Significant improvements in cognitive and motor outcomes in infancy and preschool age, but diminishing effects at school age
Ochandorena‐Acha et al. (2022) [20]	Evaluate early physiotherapy for preterm infants and parents	RCT (60)	6 sessions + home practice	Improved motor, language, and problem‐solving; high compliance linked to better outcomes
Wang et al. 2021 [21]	To examine the effects of KMC on neurobehavioral development	RCT with follow‐ups (36 KMC group)	KMC	KMC improved breastfeeding rates, weight gain, and neurobehavioral outcomes
Fan et al. (2021) [22]	Evaluate home‐based, post‐discharge early intervention for preterm infants	Prospective RCT (73 PT)	Interventions included intellectual, physical, and social training	Significant improvements in motor performance (TIMP), DQ, and physical growth
Pineda et al. (2021) [23]	Assess the SENSE program for preterm infants	RCT (70 days sensory program)	Sensory‐based interventions	Improved communication skills but increased lethargy
Soleimani et al. (2020) [24]	To assess the impact of DSC on cognitive and motor outcomes of preterm infants	RCT, assessment at 12 & 24 months	DSC	DSC improved cognitive and motor indices at 12 months but showed no significant benefits at 24 months
Lu et al. (2019) [25]	Evaluate massage therapy on motor and sensory development in children with delays	RCT (36 PT)	Massage group receive 20 min, two times every weeks for 12 weeks	Significant improvements in motor, gross motor, and sensory scores
Neel et al. (2019) [26]	Impact of MSS on neurodevelopment	RCT (200 PT)	MSS	MSS improves neural processing and motor outcomes
Mohamed et al. (2018) [27]	MSS and neurobehavioral development	RCT (40 PT)	MSS	Significant improvements in all neurobehavioral measures except state regulation
Kanagasabai et al. (2013) [28]	Effect of MSS on neuromotor development in preterm infants	RCT (50 PT)	ATVV stimuli on the neuromotor development of preterm newborns	MSS appears to promote acoustic maturation in premature babies. However, more investigation is necessary to determine the long‐term effects
Lee et al. 2018 [29]	The effects of early‐stage neurodevelopmental treatment on the growth of premature infants in the NICU	RCT (85 PT)	The neurodevelopmental treatment includes various sensory inputs, such as tactile, proprioceptive, vestibular, visual, and auditory sensations	Sensory stimulation improved weight gain and motor development
Liaw et al. (2012) [30]	NNS and facilitated tucking relieve preterm infant pain during heel‐stick procedures	RCT (34 PT)	NNS	NNS and assisted tucking are just as beneficial as normal care in lowering the pain score
Ding et al. (2019) [31]	To analyze the effect of FCC on preterm infant development and parental stress	Prospective cohort study	FCC	FCC improved weight gain, reduced hospital readmissions, and decreased parental anxiety
Lee et al. (2017) [32]	Evaluate the impact of neurodevelopmental treatment on motor development in preterm infants using the TIMP	RCT, 96 preterm babies	Neurodevelopment which includes tactile, proprioceptive, vestibular, visual, and auditory stimulation, alongside standard NICU care treatment for 15 min 4 times per week up to 40‐week postconceptional age	The intervention group showed significantly higher TIMP scores at 40 weeks compared to the control group (*p* < 0.01) and also outperformed both the control and low‐risk comparison groups
Nair et al. (2014) [33]	Evaluate developmental intervention package for low birth weight infants	Longitudinal study (821)	Stimulation program, home‐based interventions	Improved motor DQ, reduced abnormalities in motor development

Abbreviations: ATVV, auditory, tactile, visual, and vestibular; DQ, developmental quotient; DSC, developmental supportive care; FCC, family‐centered care; HR, heart rate; KMC, Kangaroo Mother Care; MSS, multisensory stimulation; NICU, neonatal intensive care unit; NIDCAP, Newborn Individualized Developmental Care and Assessment Program; NNS, nonnutritive sucking; PT, physical therapy; RCT, randomized controlled trial; RR, respiratory rate; SENSE, supporting and enhancing NICU sensory experiences; TIMP, Test of Infant Motor Performance.

The intervention components of the NLDIP were systematically identified through a comprehensive review of evidence‐based literature. This included guidelines from reputable sources such as the World Health Organization (WHO) and the NIDCAP, established models of developmental supportive care (DSC), and peer‐reviewed articles focusing on neurodevelopmental interventions for preterm infants. The majority of the included studies were systematic reviews, meta‐analyses, and RCTs.

To assess the methodological quality and risk of bias of the primary RCTs, the Joanna Briggs Institute (JBI) checklist was utilized. The assessment indicated a low to moderate risk of bias across the studies (see Table 2). Although the overall methodological quality was deemed fair, common limitations included small sample sizes and the presence of selection and detection biases in some studies. Despite these limitations, the literature consistently supported the effectiveness of developmental supportive interventions in improving neurodevelopmental outcomes in preterm infants. Based on this evidence, key intervention strategies with strong potential for clinical applicability were selected and incorporated into the NLDIP.

**TABLE 2:** JBI risk of bias checklist for primary studies.

Author (Year)	Study design	Randomization	Allocation concealment	Blinding (participants/personnel)	Blinding (outcome assessors)	Follow‐up completeness	Outcomes measured reliably	Appropriate analysis	Overall risk
Ochandorena‐Acha (2022) [20]	RCT	+	−	−	+	+	+	+	Moderate
Wang et al. (2021) [21]	RCT	+	+	+	+	+	+	+	Low
Fan et al. (2021) [22]	RCT	+	−	−	−	+	+	+	Moderate
Soleimani et al. (2020) [24]	RCT	+	−	−	−	+	+	+	Moderate
Lu et al. (2019) [25]	RCT	+	−	−	−	+	+	+	Moderate
Neel et al. (2019) [26]	RCT	+	−	±	+	+	+	+	Moderate
Mohamed et al. (2018) [27]	RCT	+	−	−	−	+	+	+	Moderate
Kanagasabai et al. (2013) [28]	RCT	+	−	−	−	+	+	+	Moderate
Lee et al. (2018) [29]	RCT	+	−	−	−	+	+	±	Moderate
Lee et al. (2017) [32]	RCT	+	−	−	−	+	+	+	Moderate
Liaw et al. (2012) [30]	RCT	+	−	+	+	+	+	+	Moderate
Ding et al. (2019) [31]	Cohort	NA	NA	NA	NA	+	+	+	Low
Nair et al. (2014) [33]	Longitudinal	NA	NA	NA	NA	+	+	±	Moderate

*Note:* Systematic reviews and meta‐analyses were excluded from this table as JBI bias checklists apply only to primary research studies. NA, Not applicable to study design; RCT, randomized controlled trial.

#### 3.1.2 Identifying/Developing the Theory

The literature identified several key models that underpin the understanding of DSC, specifically neonatal integrative developmental care model: This model emphasizes a holistic approach that integrates environmental, relational, and sensory aspects to promote optimal development in neonates. Synactive model: This framework focuses on the interactions between the infant's neurodevelopmental status and the caregiving environment. It highlights the importance of recognizing and responding to the infant's cues and stress signals. Transactional model: This model stresses the bidirectional influence between the child and their environment, including parenting behaviors, which can significantly affect the developmental outcomes of preterm infants. NIDCAP suggests the importance of family‐centered care (FCC) and cue‐based care for preterm infants [9, 11, 12]. These models guided the selection of interventions for the NLDIP, emphasizing strategies that reduce stress for both neonates and their parents.

#### 3.1.3 Development of NLDIP

The NLDIP was developed based on the well‐established evidence‐based intervention and theoretical model to provide a robust framework for delivering the early developmentally supportive intervention for supporting the neurodevelopmental needs of preterm infants. The NLDIP includes three components: The first component is standard DSC. This component includes evidence‐based practices in NICU such as nesting, swaddling, therapeutic positioning, containment, nonnutritional sucking (NNS), pain management, breast milk/breastfeeding, and environmental modifications. The second component is MSS in the NICU: This component includes age‐appropriate sensory interventions that support the development of the infant's sensory systems, which include tactile, auditor, vestibular, olfactory and gustatory, kinesthetic, and visual stimulation. The third component of NLDIP is MSS at home: This component is the extension of the age‐appropriate MSS into the home environment.

The rationale for integrating various interventions into a unified NLDIP was to address multiple domains of neurodevelopment in a holistic and coordinated manner, rather than implementing isolated strategies. This integrated approach promotes consistency, continuity of care, and enhances the overall development. Unlike previous studies that often evaluated individual interventions in isolation, the NLDIP provides a comprehensive, structured protocol with clearly defined procedures, enabling nurses to deliver standardized, evidence‐based care effectively within their routine clinical practice.

#### 3.1.4 Validation of NLDIP

The first component of the NLDIP comprises standard DSC, incorporating training modules, educational posters, instructional videos, and assessment tools for evaluating knowledge and skills. This component underwent validation through a single‐round Delphi technique involving six nursing experts and three medical professionals, achieving a content validity index (CVI) of 0.90. Expert feedback was systematically reviewed and integrated following deliberations with the coauthors to ensure the refinement and relevance of the content.

The second component of the NLDIP, focusing on MSS, underwent validation by specialized experts. Auditory and vestibular stimulation interventions were validated by experts from the field of speech and hearing, whereas tactile and kinesthetic interventions were reviewed by a neonatal physiotherapist. Visual stimulation was validated by a neonatal ophthalmic therapist, and all multisensory interventions were further assessed by a neonatal occupational therapist. Expert recommendations, such as initiating visual stimulation with bold black and white patterns after 31 weeks of gestational age, were incorporated into the protocol following consultation with the authors. The MSS interventions were deemed valid and age‐appropriate based on expert consensus.

### 3.2 Phase II: Feasibility Assessment

#### 3.2.1 Feasibility Assessment of First Component of NLDIP

The feasibility assessment of the first component of the NLDIP involved training sessions for five nursing officers over a period of 4 days, utilizing lectures, handouts, videos, and live demonstrations to equip staff with the necessary skills for implementing DSC. Following the training, a feasibility assessment revealed positive feedbacks regarding the educational materials, though challenges arose during implementation due to environmental constraints and resource availability in the NICU. Insights from the nursing officers led to valuable modifications, including the simplification of certain techniques and ensuring that necessary resources were accessible. This collaborative approach ensured the intervention package remained evidence‐based while being practical and adaptable to the NICU environment, ultimately enhancing both feasibility and effectiveness.

Testing of nesting and swaddling procedure‐identified challenges included the need for two bedsheets, the inability to swaddle sick babies, unavailability of sheets, and limited NICU space. After discussions, needed resources were arranged, and sterile bedside nesting was implemented. Positioning and containment were effective and feasible to practice, with no challenges or modifications needed. For Kangaroo Mother Care (KMC) and breastfeeding, the video was too long and was revised to focus on preparation, positioning, and monitoring, whereas the lack of dedicated KMC space and facilities was addressed by arranging chairs and beds after discussions. Pain management and NNS content were adequate, but consultant orders were required for implementation, and frequent staff rotations highlighted the need for additional training. Common pain management strategies were integrated after discussions with authorities. FCC and environmental modification content were sufficient, but modifying the NICU environment was out of the scope of nurses, although standardization check list is needed to modify the environment (noise reduction and dim light) in NICU.

#### 3.2.2 Feasibility Assessment of Second Component: MSS During Hospitalization and at Home

The second component of the NLDIP was tested on three mothers of preterm babies. This involved training mothers to provide MSS to neonates using various techniques. The training was delivered in two phases: on the first day, auditory, visual, and vestibular stimulation methods were taught and demonstrated; on the second day, massage, kinesthetic, olfactory, and gustatory stimulation were introduced.

Auditory stimulation was easy for mothers to implement, but challenges included mobile device restrictions for infection control and unfamiliarity with the Lohri song. To address this, a calm room was provided for voice recording, maternal voices were recorded for those unfamiliar with the song, sound speakers were sanitized, and mobiles were permitted after cleaning with isopropyl alcohol. Tactile stimulation through therapeutic touch was found beneficial, with mothers recommending coconut oil for massage; no modifications were made except for standardizing massage steps based on literature. Vestibular stimulation was simple and easy to follow, requiring no changes. For kinesthetic stimulation, mothers were concerned about their babies' fragility during the range of motion exercises, leading to the modification of applying gentler pressure and incorporating basic flexion and extension of extremities following expert consultation. Olfactory and gustatory stimulation initially faced restrictions on using personal items like a dupatta due to infection control; however, after discussion with the consultant, dupattas were allowed in the NICU with strict sterility maintenance. Additionally, a sterile gauze pad was used during KMC and placed near the baby when the mother was absent, whereas two drops of breast milk were introduced into the baby's mouth during nasogastric feeding.

A 1‐month pilot study was conducted to assess the feasibility of implementing the third component of NLDIP, MSS in a home setting. The findings indicated 90%–100% compliance with the intervention by mothers, with no reported difficulties in following the MSS at home. Final protocol adjustments were made based on recommendations from nursing officers, mothers, and experts (Table 3).

**TABLE 3:** Nurse‐led developmental intervention package.

The nurse‐led developmental intervention package			
First component: Standard DSC			
Commencement of interventions: From the time of admission in NICU till discharge			
Provider: Nursing officer			
Setting: NICU (main NICU, isolation, and step‐down area)			
Serial number	Intervention	Description	Duration of intervention
1	Nesting	Creating or preparing a nest‐like oval boundary around the infant using sterilized sheets in which the infant is placed	Prepared in morning shift and maintained throughout the day
2	Swaddling	This is a technique of wrapping the baby in a sheet so that the infant feels safe, secure, and contained	Prepared in each shift and maintained throughout the day
3	Containment	One hand of the caregiver is placed firmly yet gently on the head of the baby, whereas the other hand can be placed either on the lower back, buttocks, or soles of their feet while maintaining extremities	During painful procedure, positions change and unsettled baby
4	Positioning	The appropriate therapeutic positionings such as supine, prone, and side lying position are provided according to the child needs. The position is provided as per the modified infant position checklist	Therapeutic position maintained all the time in a day
5	NNS	NNS refers to the sucking opportunities provided to the infants in form of sucking own fingers, mother's breast, and gauze lollipop dipped in mother's milk	Once in every shift for 5–10 min. NNS is given after 32 weeks of GA
6	FCC	The principle of FCC is that the parents and the family are an integral part of the NICU team	During hospitalization
7	Early breast milk/breastfeeding practices	Initially expressed milk is given and initiation of breastfeeding as soon as the child develops good coordination in sucking and swallowing reflex	Expressed breast milk for alternative feeding once the baby tolerates the feeding. Demand/schedule beastfeeding
8	KMC	Continuous and prolonged skin‐to‐skin contact between baby and mother/caregivers	Minimum duration is 1 h and the maximum 6–8 h. Can be increased more than 8 h
9	Modification of the NICU unit	In this study, the modification of NICU environment includes dimming the individual unit lighting and lighting adjusted to reflect the cycle of the days especially at night. Implementing strategies to reduce the unnecessary noise and surrounding noise level of NICU kept below 50 dB	All the time in a day
10	Pain management	In this study, pain management in neonate refers to managing the neonate pain with the help of containment, 25% dextrose 0.1–0.5 mL, NNS, lollipop gauze dipped in breast milk, and swaddling during the painful procedure	During painful procedures such as heel prick, venepuncture, ROP screening

Second component: MSS during hospitalization			
Commencement of interventions: 31st week of corrected age till 37 weeks of corrected age			
Provider: Mothers of preterm babies and caregivers			
Setting: NICU (main NICU, isolation, and step‐down area)			
Criteria for MSS: Vital signs should be stable and apnea free			
Serial number	Intervention	Description	Duration of intervention
1	Auditory stimulation	Auditory stimulation is given through a recorded mother voice (lullaby) Calming music (veena, tabala, recorded lullaby) is put near the baby Maternal talk with the baby Miniature speaker provides auditory stimulation with noise level less than 50 dB Mother talking to the baby while caring and feeding	Each auditory stimulation is given for 10–15 min duration and two times in a day
2	Tactile stimulation	Tactile stimulation is given in the form of a mother therapeutic touch The tactile stimulation is done with two or three fingers with gentle pressure three times in one direction and the opposite direction on the face's temporal, frontal, periorbital, and nasal regions The mother is asked to touch the whole body during a diaper change	Tactile stimulation is given twice daily for 5–10 min and whenever she visits the NICU
3	Vestibule kinesthetic stimulation	Vestibule kinesthetic stimulation is provided by gently rocking the baby and slowly swinging the baby horizontally and vertically The mother is also asked to pull the baby vertically upward from the supine position by holding the shoulder and neck Avoid sudden jerky moments	Vestibule kinesthetic stimulation is given for 5 minutes two times a day
4	Visual stimulation	Vision sense is developed at last in neonates. To provide visual stimulation, dim light is provided, and avoid exposure to bright sunlight Initially, a black and white color with a bold pattern and strong contrast is kept 8–12 inches from the baby	Visual stimulation is given for 15–20 min and twice daily
5	Massage therapy	Coconut oil is used for massaging the baby. Massage is done using two fingers kept flat on the body, providing a long gentle stroke and massage in a circular pattern	The whole‐body massage is done for 5–10 min duration once a day
6	Kinesthetics stimulation	Simple passive flexion and extension of extremities	Passive exercise is provided once in a day for 5–10 min
7	Olfactory stimulation	Avoid strong, pungent smells (perfume, spirit, alcohol, etc.) Mother used cloth or cloth impregnated with mother's odor is kept near to the baby KMC is provided to the baby. It is an excellent source of olfactory stimulation	Twice a day for 10–15 min
8	Gustatory stimulation	Mother's breast milk is given to the baby Few drops of breast milk are put in the mouth if the child is on OG feeding	2–3 drops twice a day

Third component: MSS at home				
Commencement of interventions: First month of corrected age to 6 months of corrected age				
Provider: Mothers of preterm babies and caregivers				
Setting: Home setting				
Criteria for MSS: Stable condition				
Intervention	Duration of intervention	0–2 months	3–4 months	5–6 months
Auditory stimulation				
Light and calm music (tabala, veena, lullaby)	10–15 min, two times a day	Continue		
Make the child listen to different sounds such as squeeze toy, rattle, bell, music, high‐pitched, and low‐pitched human sound	5–10 min, two times a day		Continue	
Shake a bell or a squeaky toy over his head. Then, slowly shake it near the side of his head	5–10 min for two times a day			Continue
Tactile stimulation				
Stimulate the baby by tickling	2 min and two times a day	Continue	Continue	
Gentle coconut oil massage is done using two fingers placed flat on the body	5–10 min and two times a day	Continue	Continue	Continue
Passive stretching exercise: Flexion and extension of arms and legs	2 min and two times a day	Continue	Continue	Continue
Make the baby sit in a hot water tub with support	10–15 min in a day	Continue	Continue	Continue
Visual stimulation				
Initially, show the 4–5 black and white color paper with different patterns	2 min with a gap of 2 min and 2 times a day	Continue		
Use alternative colors on alternative days				
Colorful, medium‐sized, and usually sound‐making toys are hung about 12–15 inches above the crib	All the time	Continue	Continue	
Preferably bright red, yellow, and green colors				
Hold a mirror 7–8 inches away from his eyes and show them their faces	2 min for two times a day		Continue	Continue
Vestibule kinesthetic stimulation				
Gentle bouncing of the baby	2 min, 3 times a day	Continue	Continue	Continue
Horizontally slowly move the whole body	2 min for 3 times a day	Continue	Continue	Continue
When the baby is on supine, vertically lift the baby by holding the baby's head and back	2 min for 2 times a day	Continue	Continue	Continue
Walk around while holding the baby	2 min for 2 times a day	Continue	Continue	Continue
Olfactory and gustatory stimulation				
Maintain skin to skin contact (KMC)	Minimum 1 hours	Continue		
Mothers are asked to give exclusive breastfeeding till 6 months of life	Demand feeding	Continue	Continue	Continue
Olfactory stimulation includes, for example, fruity smell, floral fragrance, and the mother's chest	1 min for two times a day	Continue	Continue	Continue
Avoid perfume odors and pungent smell	All the time	Continue	Continue	Continue

Abbreviations: DSC, developmental supportive care; FCC, family‐centered care; KMC, kangaroo mother care; MSS, multisensory stimulation; NICU, neonatal intensive care unit; NNS, nonnutritive sucking; OG, orogastric feeding; ROP, retinopathy of prematurity.

### 3.3 Phase III: Implementation

A training program was developed for a NICU staff to implement the NLDIP, emphasizing the importance of DSC principles. A training program for 55 NICU nursing officers at All India Institute of Medical Sciences (AIIMS), Jodhpur, took place from December 20, 2022, to January 21, 2023. The training program consists of lectures and discussions with powerpoint (PPT), distribution training module, video teaching, hands‐on training and redemonstration. Each nurse received 3–3.5 h of training, each session lasting for 30–40 min. Small groups of 4–5 nurses were trained per session to accommodate shifts. Standard DSC was categorized into five modules. Training sessions were taken for 2–3 days to complete each module, followed by 2–3 days for observing practice compliance, during which any mistakes were corrected. A pretest and posttest knowledge and skills were assessed, which showed adequate knowledge and skills in the implementation of standard DSC in NICU by nursing officers [34].

### 3.4 Phase IV: Evaluation

A dry run was conducted after training nursing officers in DSC. Held over a week, we assessed the compliance and feasibility of the NLDIP. Ten mothers of preterm infants (31 weeks gestation) participated in the dry run to evaluate adherence to MSS. Nursing officers provided standard care to all 10 infants, whereas mothers underwent 2 days of training (using PPTs, videos, and demonstrations) on MSS. They then demonstrated the techniques and after ensuring them acquiring adequate technique, mother administered MSS to their infants over 5 days.

The dry run showed most interventions were feasible, except for pain management and environmental modification. The testing phase and dry run demonstrated good compliance with interventions such as nesting, swaddling, positioning, containment, KMC, breast milk/breastfeeding, FCC, and NNS, making them viable for NICU practice. These were included in the final supportive care package. Controlling NICU noise and light levels was challenging, and dextrose for pain management was not consistently used due to clinical reasons. NNS was also difficult to implement consistently during the painful procedure, as mothers were not always present, leading to reliance on routine pain management practices. Still, both were included in the package, but monitoring their implementation in the NICU was emphasized.

The MSS interventions showed good compliance and feasibility among mothers of preterm infants. As a result, all auditory, tactile, visual, vestibular‐kinesthetic, and olfactory‐gustatory stimulation interventions were retained in the final protocol, though visual stimulation showed low rates of compliance.

The second dry run was conducted to evaluate the compliance and noncompliance rates for the NLDIP. Nesting, swaddling, positioning, containment, KMC, and breastfeeding achieved 100% compliance. KMC was practiced for an average of 2.5–3.5 h per day, 4.7 h per day. Pain management had a compliance rate of 60%, with only nesting, swaddling, and containment provided during painful procedures as dextrose was withheld temporarily due to clinical reasons. Environmental modifications, such as controlling noise and light, proved challenging, as noise levels often exceeded 50 dB, and bright light was difficult to regulate in the NICU. Auditory, tactile, vestibular, and kinesthetic stimulations were fully complied. However, visual stimulation had only 60% compliance. Olfactory stimulation shows 80%–90% compliance rate, with some mothers unwilling to follow the protocol, whereas gustatory stimulation (breast milk) reached 100% compliance. The researcher observed during the evaluation phase that consistent implementation of NLDIP requires compliance check and motivation for both nursing officers and mothers.

## 4 Discussion

In this study, a theory‐driven and evidence‐based NLDIP was developed based on the MRC framework. The rigor and efficacy of the intervention were enhanced by using the development phase of the MRC framework. Our study supports the usefulness and feasibility of the MRC framework for guiding the development of a NLDIP for preterm babies to enhance neurodevelopmental outcomes. This article describes the design of our intervention and process of development and validation of NLDIP. A detailed scoping review was done to select the evidence‐based DSC interventions. By incorporating evidence‐based developmental care strategies, this intervention package empowers nurses and mothers to provide structured and individualized care to preterm infants, thereby enhancing their overall growth and neurodevelopmental outcome. A key innovation of the present study is the nurse‐led delivery and integration of the developmental intervention package within routine NICU care. Although components of DSC have been previously implemented in neonatal settings, they are often delivered in a fragmented manner by physicians, occupational therapists, or multidisciplinary teams. In contrast, this study uniquely positions nurses, not only as caregivers but also as central agents in delivering a structured, evidence‐based intervention. By training and empowering nurses to administer the full package of interventions, the approach enhances continuity, accessibility, and sustainability of care. This integration into daily nursing practice represents a practical and feasible practice, particularly relevant for resource‐limited settings.

The successful validation of this intervention package underscores its potential for widespread implementation in NICUs. Nurses, as frontline caregivers, play a pivotal role in neonatal care, and equipping them with a structured developmental care framework can enhance their ability to support preterm infants effectively.

The author agrees that pain management and environmental modifications posed significant implementation challenges. The author proposed prospective strategies such as training of nurses, mothers, and residents for nonpharmacological pain management strategies and development of checklist for environmental modifications. Additionally, we need good collaboration with hospital administration to ensure a supportive physical environment (noise reduction and dim lighting) for preterm babies in NICU.

The researcher believes that standard DSC combined with MSS during hospitalization and beyond the hospitalization, might be more beneficial for preterm babies to have intact survival. The feasibility assessment, piloting phase and evaluation of the intervention package lay a foundation for future large‐scale randomized control trial to evaluate the effectiveness of NLDIP on neurodevelopment outcomes of preterm babies.

### 4.1 Ethical Consideration

Ethical permission was obtained from the institutional ethics committee of AIIMS, Jodhpur (AIIMS/IEC/2022/3932). Informed consent was obtained from the nursing officers and mothers of preterm babies during the implementation and evaluation of NLDIP.

## 5 Strength and Limitation

This study utilized the scientific evidence‐based interventions to develop the age‐appropriate NLDIP. One of the key strengths of this study is its rigorous methodology, including the systematic development and validation of the intervention package. A Delphi technique was adopted to validate the interventions from experts in the specific area of NLDIP. We used all four phases of MRC framework to develop and validate the NLDIP. The successful implementation of the NLDIP for preterm neonates requires active collaboration between nurses and mothers. In our setting, the practice of FCC facilitates this collaboration by enabling mothers to stay with their infants in the same room during NICU admission. This model promotes maternal involvement in the daily care of their preterm infants, enhancing adherence to the intervention package and reinforcing developmental support beyond routine nursing activities.

However, certain limitations must be acknowledged. The study was limited to a single NICU setting which may affect the generalizability of the findings. Other limitation of the study was the low compliance (60%) with the visual stimulation component of the intervention. This was primarily due to the clinical condition of preterm neonates, such as ongoing phototherapy, illness, or prolonged sleep states, which limited opportunities for visual engagement. Although mothers reported no difficulty in performing the activity, the infants' responsiveness was often low. Despite this, the component was retained due to strong evidence supporting its role in visual tracking and cortical development. Future implementation may benefit from flexible scheduling and proactive nurse‐initiated visual engagement using black‐and‐white‐contrast visual aids.

Furthermore, the evaluation of the NLDIP was conducted with a small sample size over a short duration, primarily to assess compliance and adherence to the intervention components. Additionally, as the primary objective of this study was the development and validation of the NLDIP, it was not designed to evaluate its effectiveness on neurodevelopmental outcomes. Future studies with larger samples and longer follow‐up periods are needed to assess the impact of the intervention on developmental trajectories.

Further, consistent implementation requires frequent observation and compliance checking of intervention package in NICU. Additionally, there is a need of stakeholder's support, adequate training, and resources to implement NLDIP in NICU. Additionally, scaling up the implementation of this package across diverse healthcare settings can provide valuable insights into its adaptability and effectiveness in different contexts. Future research should also investigate the cost‐effectiveness of this approach to support its integration into routine neonatal care.

## 6 Conclusion

This study provides strong evidence supporting the development and validation of a NLDIP for preterm neonates. The NLDIP offers a structured approach to improving the quality of care in the NICU, fostering an environment that supports the holistic development of preterm babies. By empowering nurses and mothers with structured intervention and training, this intervention package has the potential to enhance neurodevelopmental outcomes and improve overall neonatal care. Further research and widespread implementation efforts are essential to maximize the benefits of this approach for preterm infants globally.

## Author Contributions

Raghu V. A.: Planned and prepared a conceptualization of study, identification of evidence bases and theories, validation of NLDIP, and implementation and evaluation of NLDIP. Manju Vatsa: Contributed to the design and implementation of the research, to the analysis of the results, and to the writing of the manuscript. Neeraj Gupta: Contributed to the design and implementation of the research, to the analysis of the results, and to the writing of the manuscript. Hansraj: Contributed to the implemention and feasibility testing of NLDIP. Mr. Ramesh Choudhary: Contributed to the implemention and evaluation of NLDIP.

## Ethics Statement

Ethical permission was obtained from the institutional ethics committee of AIIMS, Jodhpur (AIIMS/IEC/2022/3932). Informed consent was obtained from the nursing officers and mothers of preterm babies during the implementation and evaluation of NLDIP.

## Conflicts of Interest

The authors declare no conflicts of interest.

## Data Availability

The data that support the findings of this study are available from the corresponding author upon reasonable request.
